# The inappropriateness of brain MRI prescriptions: a study from Iran

**DOI:** 10.1186/s12962-021-00268-6

**Published:** 2021-03-05

**Authors:** Zahra Kavosi, Abouzar Sadeghi, Farhad Lotfi, Hedayat Salari, Mohsen Bayati

**Affiliations:** 1 Health Human Resources Research Center, School of Management and Information Sciences, Shiraz University of Medical Sciences, Almas Building, Alley 29, Qasrodasht Ave, Shiraz, Iran; 2 Health Policy and Management Department, Bushehr University of Medical Sciences, Bushehr, Iran

**Keywords:** Magnetic Resonance Imaging, Health Expenditures, Medical Overuse, Unnecessary Procedures, Neurology, Prescriptions

## Abstract

**Background:**

Inappropriate prescriptions can lead to adverse consequences for patients. It also imposes excessive cost on the patients, payers and health systems. The current study aimed at estimating the rate of inappropriate brain Magnetic Resonance Imaging (MRI) prescriptions and their financial burden in Iran.

**Methods:**

Using systematic stratified sampling method, this cross-sectional study recruited 385 participants from three public teaching hospitals in Shiraz, Iran. Demographic information, questions related to brain MRI prescription and its indications checklist were collected using study-specific data collection tools. The completed indications checklist was compared to the appropriateness status table of indications and scenarios to detect the percent of the appropriateness of prescriptions.

**Results:**

About 21 percentage of total brain MRI prescriptions are inappropriate. Previous treatment, number of referrals to physician, having other diagnostic tests and the applicant of MRI (P < 0.01) had significant relationships with prescription appropriateness. The estimated financial burden of inappropriate brain MRIs in Shiraz teaching hospitals was 99,988 US dollar in 2017.

**Conclusions:**

More than one-fifth of brains MRIs were inappropriate (i.e. prescriptions without medical indications). It caused 99,988 United States Dollar (USD) financial burden which is 17 times that of Iran's Gross Domestic Product (GDP) per capita. To better allocate resources for the provision of MRI services to health system, rationing policies for controlling moral hazard and reducing provider induced demand can be helpful.

## Background

Neurological diseases are one of the common causes of death and physical and mental disabilities in different societies. Neurological diseases are disorders that influence the central and peripheral nervous system including brain, spinal cord, and nerves such as cerebrovascular disease [1]. Early and precise diagnosis of brain and neurological disorders and damages will assist in the better and fast treatment of those illnesses. Magnetic Resonance Imaging (MRI) have important roles in diagnosing these illnesses [2]. In addition to assisting in the early diagnosis of illness, imaging prevents more aggressive measures for treatment [3]. In this respect, one application of MRI is diagnosis of brain and neurological illnesses [4].

It is stated that only less than half of MRIs prescribed by neurologists were appropriate [5]. Despite the significant effects of MRI in diagnosing diseases, unnecessary provision of it can impose financial burden on patients, payers and healthcare systems [6, 7].

There is substantial growth in MRI acquisition and utilization in Iran [8]. According to a local research in Iran, about 80 percentage of demanded MRIs by physicians for the patients suffering from headache had normal results. It shows that MRI prescriptions in many cases are illogical and inappropriate [9]. According to Salari et al. [10], 167 lumbar spine MRI prescriptions among 300 prescriptions were inappropriate, and the cost of these inappropriate prescriptions was estimated at 7178 US dollar. So, health economists and policy makers believe that evidence-based and appropriate use of these services reduces health services costs and promotes services quality [5].

According to the last published statistics in Iran in 2018, total health expenditure (THE) was about 39 billion United States Dollar (USD) (8.7 percentage of Gross Domestic Product (GDP)), which was 484.3 USD in terms of per capita. Public spending, including social insurance and government budget, accounted for 45.9 percentage of THE. Out of pocket payments as the main source of health financing accounted for 35.8 percentage of THE [11]. In an unpublished study in Iran, it is reported that MRI services cost that covered by insurance organizations (both social and private) was about 6.8 million USD in 2018 [12]. Anyway, as mentioned by other researchers, advanced diagnostic procedures such as MRI are specified as main drivers of the health care expenditure [13, 14].

The present study aimed to evaluate the level of brain MRI inappropriate prescriptions and their financial burden in public teaching hospitals in Shiraz in 2017**.**

## Methods

This research is a cross-sectional study that was carried out in 2017 to evaluate the level of inappropriate prescriptions of brain MRIs and their financial burden in teaching hospitals of Shiraz University of Medical Sciences.

Using systematic stratified sampling method, this research recruited 385 participants from public teaching hospitals in Shiraz, Iran.

The total sample size was proportionally allocated to three public hospitals namely, Namazi, Faghihi, and Chamran, which were the only MRI services providers in Shiraz’s public sector, according to their volume of MRI services.

To select participants in each hospital, the waiting list of patients was obtained from the hospitals’ MRI departments, and the participants were randomly selected using random number tables.

To collect data, one nurse attended in patients' readiness rooms and examined the prescriptions of the patients who were selected previously through a systematic sampling from the waiting list of MRI. We exclude the prescriptions that did not have a patient history or MRI prescriptions that have not registered.

The selected participant's information was collected through a data collection form. This form consisted of two parts. The first part was about patients' demographic information and the questions related to prescription and the second part was the brain MRI prescription indications checklist. We used the checklist that was developed by Salari et al. [15].

The data obtained from data collection forms was compared to the appropriateness status table of indications and scenarios to detect the percentage of the prescriptions’ appropriateness. Salari et al. developed these indications and scenarios appropriateness status table. In that consensus-based study, experts in brain disorders (neurosurgeons and neurologists) scored the scenarios according to Rand Appropriateness Method. The scenarios were classified into the categories of appropriate, uncertain and inappropriate [15].

To estimate the number of inappropriate MRI prescriptions, the estimated rate of inappropriateness of brain MRI was multiplied by total brain MRI services provided by public hospitals in Shiraz. Finally, by multiplying the number of inappropriate prescriptions by the brain MRI procedure's tariffs for the patient and insurer in 2017 the overall imposed financial burden was estimated. It should be noted that in the current study the financial costs of inappropriate MRI were estimated and the comprehensive economic burden of them was not studied. For converting Iranian Rial to USD exchange rate reported by the central bank of Iran was used [16]. Statistical analysis was performed by Stata 14.

## Results

About 60 percentage of patients were female and 96 percentage have basic health insurance. The education level of patients was elementary and secondary (33.2), academic (28.7), high school diploma (25.5) and illiterate (13.5), respectively. 37 percentage of patient had complementary health insurance.

Table 1 shows descriptive statistics of the appropriateness status of brain MRI in studied patients and the relationship between examined patients’ socio-demographic variables with the appropriateness of brain MRI descriptions. The results indicate that generally 21.6 percentage of total prescriptions are not appropriate.

**Table 1:** Appropriateness of brain MRI prescriptions based on patients’ socio-demographic variables in Shiraz public hospitals in 2017

Overall		With indication		Without indication		Total		P-value
		Frequency	Percent	Frequency	Percent	Frequency	Percent	
		302	78.45	83	21.55	385	100	
Gender	Male	116	76.82	35	23.18	151	100	0.535
	Female	186	79.5	48	20.5	234	100	
Education level	Illiterate	39	75	13	25	52	100	0.094
	Elementary & secondary	105	82	23	18	128	100	
	High school diploma	82	83.67	16	16.33	98	100	
	Academic	76	71	31	29	107	100	
Job	Employee	53	76.81	16	23.89	69	100	0.949
	Worker	38	79.17	10	20.83	48	100	
	Housewife	126	79.24	33	20.76	159	100	
	Self-employed	65	76.47	20	23.53	85	100	
	Other	20	83.34	4	16.66	24	100	
Basic insurance status	Not insured	11	84.61	2	15.39	13	100	0.743
	Insured	291	78.22	81	21.78	372	100	
Insurer	Social security organization	128	81	30	19	158	100	0.211
	Iran health insurance organization	143	78	40	22	183	100	
	Armed forces insurance organization	10	66.6	5	33.4	15	100	
	Other insurers	10	62.5	6	37.5	16	100	
Complementary insurance coverage	Yes	81	77.88	23	22.12	104	100	0.921
	No	221	78.64	60	21.36	281	100	

Table 2 also indicates the mentioned statistics based on clinical variables. Results demonstrated that previous treatment, number of times of visit, having other diagnostic tests and the applicant of MRI (P < 0.01), type of treatment and the specialty of the physician (P < 0.1), had significant relationships with prescriptions appropriateness.

**Table 2:** Appropriateness of brain MRI prescriptions based on clinical variables in Shiraz public hospitals in 2017

Overall		With indication		Without indication		Total		P-value
		Frequency	Percent	Frequency	Percent	Frequency	Percent	
		302	78.45	83	21.55	385	100	
Having previous treatment	Yes	115	68.86	52	31.14	167	100	0.001
	No	187	85.78	31	14.22	218	100	
Type of treatment	Drug	137	84.05	26	15.95	163	100	0.068
	Rest	6	75	2	25	8	100	
	Surgery	42	95.45	2	4.55	44	100	
	Others	2	66.67	1	33.33	3	100	
Treatment duration	Less than 1 Year	59	78.67	16	21.33	75	100	0.112
	1–5 years	73	86.90	11	13.10	84	100	
	More than 5 Years	55	93.22	4	6.78	59	100	
Number of times of visit	First time	138	72.25	53	27.74	191	100	0.001
	Second times	68	75.56	22	24.44	90	100	
	Third times or more	96	92.3	8	7.7	104	100	
Having other diagnostic tests^a^	Yes	159	85.48	27	14.52	186	100	0.001
	No	143	71.86	56	28.14	199	100	
Applicant	Physician	279	80.63	67	19.37	346	100	0.001
	Patient	6	37.5	10	62.5	16	100	
	Physician & patient	17	73.91	6	26.09	23	100	
Referral center	Public hospital	194	79.83	49	20.17	243	100	0.254
	Private hospital	20	66.67	10	33.33	30	100	
	Private office	88	78.57	24	21.43	112	100	
Specialty of the physician	Neurosurgery	78	80.41	19	19.59	97	100	0.064
	Neurologist	123	67.95	58	32.05	181	100	
	Other	101	94.39	6	5.61	107	100	

^a^CT scan, electroencephalography, etc

Among 16,200 brain MRIs that were done in studied (three) hospitals in 2017, 21.6 percentage, it means 3500 cases were brain MRIs without indication, among which 2240 cases (64 percentage) were with injection and 1260 cases (36 percentage) were with and without injection. The tariffs of doing a brain MRI procedure without injection was 22.7 USD and the tariffs of doing a brain MRI procedure with and without injection was 39 USD in 2017.

So the financial burden of inappropriate brain MRIs on the patients and insurers for “without injection”, “with and without injection” and all MRIs were 50 848, 49,140 and 99,988 USD, respectively (Table 3).

**Table 3:** Financial burden of inappropriate brain MRIs in public hospitals in Shiraz, 2017

	Number of inappropriate brain MRIs	Tariffs of one MRI	Total cost (USD)
Without injection	2240	22.7	50,848
With and without injection	1260	39	49,140
Total	3500		99,988

## Discussion

Inappropriate prescriptions for MRI services can lead to an adverse outcome for patients and imposes an excessive cost on the patients and health systems. So the appropriateness of brain MRI prescriptions, its related factors and financial burden were investigated in this study.

Findings show that 21.6 percentage of brain MRI prescriptions were without indication (inappropriate). Researches that mainly assessed the brain MRI appropriateness are few, so other MRI services are also compared with our findings. Piersson et al. [17] reported that about 19 percentage of brain MRIs in a single center in Ghana were inappropriate. It seems the use of MRI procedure in Iran is similar to that of the United States of America (USA), which has a very costly health system; because Lehnert & Bree (2010) found that 26 percentage of MRI and Computerized Tomography (CT) scan prescriptions in the USA are inappropriate [18]. Oikarinen et al. [19] indicated that 7 percentage of MRIs done in an academic hospital in Finland were inappropriate. Saadat et al. [20] found that 17.2 percentage of conducted MRIs in private centers in Tehran were normal that 9.8 percentage of them were for headache examinations. Barzin et al. demonstrated that 81 percentage of requested MRIs by physicians for patients suffering from headaches in an educational hospital in Sari city had normal results [9]. Sheehan et al. [21] reported that 45 percentage (106 cases) out of 237 prescribed MRIs for shoulder in a department of veterans affairs tertiary care hospital were inappropriate. In recent research in Ontario, the inappropriate rate of hip MRI is estimated at 32.1 percentage [22].

The Comparison also shows that there are different results on different MRI services (all MRI, brain, shoulder, hip, …). There are also different inappropriate rate of MRI prescriptions across different countries and settings associated with their health system structure and policies to control physician and patient behavior. However, it seems these differences should be mainly explained by different methodology, indications criteria, and instruments used by different researchers.

Analytic findings show that there are no significant relationships between socio-demographic variables and brain MRI appropriateness. However, several clinical factors have a significant relationship with the appropriateness of brain MRI.

We found that having previous treatment and other diagnostic tests are related to brain MRI’s appropriateness. Manta [22] showed that having no previous radiographic examination was the most predictor of inappropriate prescription of hip MRI in Ontario.

Also, Sheehan et al. [21] showed that ultrasound could be a cost-saving substitution for 66 percentage of shoulder MRIs.

Finding also indicates that 27.74 percentage of 191 patients at the first visit to the physicians were without indication for brain MRI. We also found the more number of times a patient visits a physician, the less likely the inappropriateness of brain MRI. Patients with more visits to doctors likely have chronic and severe symptoms [23], so their MRIs probably are necessary and appropriate. It also can be explained by defensive medicine. In the first visit, physicians reduce and compensate for malpractice liability risk and apply more tests and treatments, which may be unnecessary [24].

One of the main results is that the primary applicant of MRI has a significant relationship with brain MRI’s appropriateness. About 19 percentage of prescriptions requested primarily by physicians were inappropriate, but on the contrary, more than 60 percentage of brain MRIs requested primarily by patients were inappropriate. Patients usually appeal diagnostic tests e.g., MRI from their physician; however, many times, their request is unnecessary. Although it likely increases patients’ satisfaction and health outcomes, it wastes health system resources and maybe increases the side effects of these tests [25]. It is also related to the consumer moral hazard concept, resulting from information asymmetry between purchasers and patients [26].

Some macro factors which can affect on volume of unnecessary diagnostic tests and interventions that we could not assess their effect. For example, Andrade et al. found that more supply of imaging services (imaging centers, MRI machines, radiologists) by itself increase the demand for such services [27]. This phenomenon can be explained by provider induced demand or more accessibility to these services.

The present study showed that the financial burden resulting from inappropriate brain MRI prescriptions in 2017 was 99,988 USD in Shiraz public hospitals, which is about 17 times of Iran's GDP per capita in that year (5680 USD) [28]. This financial burden is related to one type of MRI services (brain MRI), in one city and only public centers. Considering all the mentioned factors, there is a noticeable financial burden of inappropriate MRI services in Iran. It should be asserted that according to a national study in 2018, there were 276 MRI machines in Iran (about 3.5 per one million population) [29].

There are few studies with a focus on Brain MRI. Moreover, different studies have utilized different tools for checking the appropriateness of MRI services, and finally, there are different approaches to estimate the financial/economic burden. As we estimated only the direct financial burden of inappropriate brain MRI services, it is not compared with other areas.

A major limitation of the current study is that there are not clear statistics about the utilization of brain MRI in Iran, so we cannot estimate the overall financial burden of brain MRI in Iran. We only estimated the financial burden of brain MRI in public hospitals in Shiraz. Another main limitation is that we calculated the only direct medical financial costs of inappropriate brain MRI and comprehensive economic burden of them (such as transportation cost, food cost, productivity loss and intangible cost) was not studied. Moreover, the lack of similar studies on brain MRI, relevant factors and its financial burden was another limitation.

## Conclusions

About one-fourth of brains MRIs were inappropriate. It caused 99,988 USD financial burden which is 17 times of Iran's GDP per capita. To better allocation of health system resources for the provision of MRI services, rationing policies for controlling consumer moral hazard and reducing provider induced demand can be helpful.

## Data Availability

The data used in this study are not publicly available because the participants were promised that the raw data would remain confidential. However they are available from the corresponding author on reasonable request.

## Abbreviations

CT: Computerized tomography
GDP: Gross Domestic Product
MRI: Magnetic Resonance Imaging
THE: Total Health Expenditure
USA: United Stated of America
USD: United States Dollar
